# The complete chloroplast genome of *Rhamnus crenata* Siebold & Zuccarini (Rhamnaceae)

**DOI:** 10.1080/23802359.2021.1945502

**Published:** 2021-07-26

**Authors:** Junfeng Wang, Silin Yang

**Affiliations:** Key Laboratory of Biodiversity Conservation in Southwest China, State Forestry Administration, Southwest Forestry University, Kunming, China

**Keywords:** *Rhamnus crenata*, chloroplast genome, phylogeny

## Abstract

*Rhamnus crenata* Siebold & Zuccarini is deciduous shrub or small tree plant that widely distributed in temperate and tropical regions of East to Southeast Asia. It has a chloroplast genome structure similar to that of other species of *Rhamnus*. It is 160,454 bp in size, including a large single-copy (LSC) region of 88,884 bp, a small single-copy (SSC) region of 18,734 bp and a pair of inverted repeats (IRs) of 26,418 bp. A total of 122 genes were annotated, including 80 protein-coding genes, 38 transfer RNA (tRNA) genes, and 4 rRNA genes. The overall GC content is 30.71%. Phylogenetic analysis shows that *R. crenata* is clustered with *R. taquetii* and *R. globosa.*

*Rhamnus crenata* Siebold & Zuccarini (Rhamnaceae) is a deciduous shrub or small tree plant that widely distributed in mountain forests or thickets in temperate and tropical regions of East to Southeast Asia (Chen and Schirarend 2007). It has been used for a variety of ornamental purposes including hedge, privacy screen, windbreak and backdrop for perennial plantings. In this study, we characterized the complete chloroplast genome sequence of *R. crenata* (DDBJ accession number: LC635131) which would be helpful for phylogeny genetics research of Rhamnus and Rhamnaceae.

Fresh leaves of *R. crenata* without disease were collected from Ningbo, Zhejiang Province, China（N 29°44′14ʺ, E 121°06′45ʺ, 590 m）and dried immediately using silica gel in the field. The voucher specimen was deposited in the Herbarium of Southwest Forestry University (SWFC) (http://bbg.swfu.edu.cn, Bin Tian, tianbin@swfu.edu.cn) under collection numbers of WJF2019003. We used DNeasyTM Tissue Kit (Qiagen, Hilden, Germany) to isolate total genomic DNA from dried leaves, and DNA samples were properly stored at Key Laboratory of Biodiversity Conservation in Southwest China, State Forestry Administration, Southwest Forestry University. The genomic paired-end (PE150) sequencing was performed on an Illumina Hiseq 2000 instrument (Illumina, Inc., San Diego, CA, USA). The chloroplast (cp) genome was assembled using the program Getorganelle (Jin et al. 2020). Annotation was performed using PGA (Qu et al. 2019) and visualized with OGDRAW (Greiner et al. 2019), coupled with manual correction for start and stop codons of protein-coding genes.

The cp genome of *R. crenata* resulted in a typical circular quadripartite structure of 160,454 bp length, with a LSC region of 88,884 bp, a SSC of 18,734 and two inverted repeats (IRa and IRb) each with a length of 26,418 bp. A total of 126 genes were annotated, including 84 protein-coding genes, 34 transfer RNA (tRNA) genes, and 8 rRNA genes. The overall GC content is 30.71%.

To verify the phylogenetic position of the newly obtained plastome of *R. crenata* and further clarify the evolutionary relationships within family Rhamnaceae, phylogenetic analyses based on total 26 complete cp genomes performed. After cp genomes was aligned via mafft (v.7.407) software (Katoh and Toh 2010) and removed gaps with Gblocks (Castresana 2000), Maximum Likelihood (ML) phylogenetic tree was constructed in the iqtree v.1.6.12 software (Nguyen et al. 2015) with bootstrap 1000 replicates and GTR + I model. The ML tree shows very clear results and most of the nodes had 100% bootstrap that *Rhamnus crenata* was belonged to *Rhamnus* and clustered with *R. taquetii* and *R. globosa.* (Figure 1). Our findings provide a foundation for further investigation of chloroplast genome evolution in Rhamnaceae.

**Figure 1.**
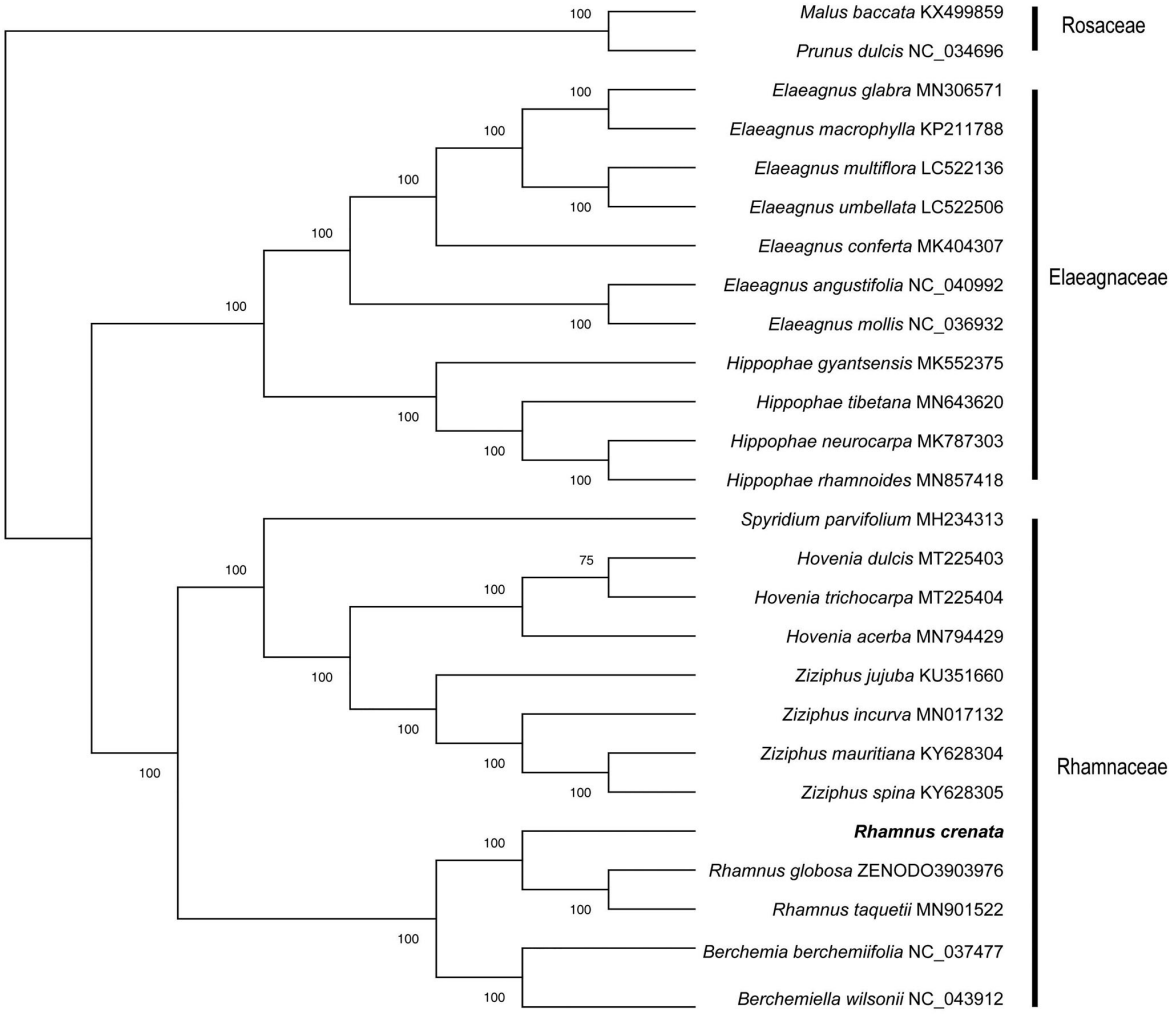
The maximum likelihood (ML) phylogram.

## Data Availability

The genome sequence data that support the findings of this study are openly available in The DNA Data Bank of Japan (DDBJ; http://www.ddbj.nig.ac.jp) under the accession no. LC635131. The associated BioProject, SRA and Bio-Sample numbers are PRJDB11553, DRR288488, and SAMD00298364, respectively.
